# Role of stressful life events and personality traits on the prevalence of wish to die among French physicians

**DOI:** 10.3389/fpubh.2024.1244605

**Published:** 2024-01-23

**Authors:** Emmanuel Diaz, Diana Abad-Tortosa, Maha Ghezal, Josephine Davin, Jorge Lopez-Castroman

**Affiliations:** ^1^Department of Psychiatry, Nîmes University Hospital (CHU), Nîmes, France; ^2^IGF, CNRS-INSERM, University of Montpellier, Montpellier, France; ^3^CIBERSAM, Madrid, Spain; ^4^Department of Signal Theory and Communication, Universidad Carlos III, Madrid, Spain

**Keywords:** occupational stress, personality, physicians, psychological stress, suicidal ideation, suicide

## Abstract

**Background:**

Suicide rates are higher among physicians than in the general population. We aimed to investigate the role of stressful life events (related or not to work conditions) and personality traits on wish to die, a proxy measure of suicidal ideation.

**Methods:**

This cross-sectional study took place in France from March 2018 to September 2018. Physicians completed an online questionnaire. A multiple logistic regression model estimated factors associated with wish to die. Moderated moderation models were used to assess the effect of personality traits on the relationship between stressful events and wish to die.

**Results:**

1,020 physicians completed the questionnaire. Most (75%) had endorsed a work-related stressful event and one in six (15.9%) endorsed a wish to die the year before. Wish to die was associated with burnout (OR = 2.65, 95%CI = 1.82–3.88) and work-related stressful events (OR = 2.18, 95%CI = 1.24–3.85) including interpersonal conflicts, harassment and work-overload. Emotional stability was the only personality trait associated with wish to die in the logistic regression (OR = 0.69, 95%CI = 0.59–0.82). In moderation models, we observed a significant interaction involving three personality traits—emotional stability, extraversion, and agreeableness—along with gender, influencing the impact of stressful events on the wish to die.

**Limitations:**

Our study is limited by the impossibility to control for risk factors associated with suicide like psychiatric comorbidities.

**Conclusion:**

Work-related stressful events significantly contribute to the manifestation of a wish to die among physicians. The impact of stressful events on the wish to die is moderated by factors such as gender and personality traits, including emotional stability and extraversion. These results are overall consistent with prior studies concerning the risk of burnout and suicide among physicians.

## Introduction

Suicidal behavior is a major public health concern worldwide. In the literature there is increasing interest in vulnerable groups like health workers, including physicians (1–4). Nowadays, health programs with specific preventive strategies targeting physicians are emerging in some countries (1, 2). A recent systematic review and meta-analysis has found that physicians are at a high risk of suicide with an overall Standardized Mortality Ratio of 1.44 (5). The lifetime prevalence of suicidal ideation (SI) varies from 20% (6, 7) to 50% (8) and 1.6% of physicians attempt suicide at least once (8). Indeed, physicians are more exposed to mental illness than the general population, notably for depressive-anxiety disorders and substance use disorder (2). More precisely, female physicians are associated with higher risk ratios for suicide, suicide attempts (SA) and SI than men (2, 4–6, 8–11). Furthermore, certain specialties such as general practitioners, psychiatrists, interns, anesthesiologists, general surgeons, obstetricians and orthopedists tend to be associated with a higher suicide risk (5). In 2016, a systematic review of medical students found that 11% of them had SI in their final year (12) [a prevalence of SI up to 18% in some studies (13) or even 30% of the sample (14)]. Moreover, 4 to 7% of medical students have already attempted suicide (13, 14). Indeed, the prevalence of depressive symptoms is estimated at around 20–30% among these students (15, 16), the difficulty for them to reconcile their studies with their personal life being associated with SI (17).

Generally speaking, physicians are exposed to high levels of professional stress: high workloads with long hours (1, 18–20), sleep disruptions (20, 21), shift-related and on-call-related stress symptoms (1, 22), an aggressive administrative working environment (19), lack of resources (1, 19), poor social support (19, 23) and violence (24). These can all lead to difficulties in sharing personal and professional responsibilities (1, 20) with an unfulfilled work-life balance (20, 25).

Several studies in the literature have established a link between the particularly high suicide risk among physicians and their working conditions (8, 17, 26). More precisely, harassment (26), conflicts with co-workers (22, 26), and lack of cohesive teamwork with poor social support (22, 26, 27) have been specifically associated with suicide risk. Indeed, these elements can lead to a poor quality of life (24) and less job satisfaction (28) associated with SI (29). However, many health professionals are loath to seek help for fear of social and professional discrimination (2, 30–33).

The increase in suicidal risk among doctors could be linked to variables other than working conditions themselves, such as personality traits. Indeed, specific personality traits are correlated with an increased risk of suicide, with some being more prevalent among doctors. A comparative analysis is necessary to further explore this association. On one hand, hopelessness (34), neuroticism (34–36), agreeableness (34–36), openness (36), anxiety (37) and extraversion (34–37) are involved in suicide risk. On the other hand, even if there is no evidence for one specific physician personality (38), obsessive compulsive (39), dysthymic (39), achievement-oriented (40), conscientious (40), introverted (41), and anxious (41) traits may be more prevalent among physicians than in the general population (42). These traits are probably adaptive to a certain extent but they could also mediate vulnerability to suicide due to working conditions. Very few studies have investigated the suicidal risk specifically associated with physicians’ personality traits. An association between neuroticism and suicide risk among physicians has already been suggested (43). Indeed, neuroticism is associated with high levels of perceived job stress and depression or anxiety symptoms among physicians (44). At the opposite, openness, agreeableness and extraversion have already been associated with well-being at work and differ according to the medical specialty (45).

Research is thus required to identify specific suicide risk factors among physicians in order to improve targeted prevention in their workplace (46). In this cross-sectional study, we contacted physicians from all specialties in France with the aim of examining the association of stressful life events, especially work-related events, on a proxy measure related to suicide risk: the wish to die. Additionally, we examined the role of personality characteristics as moderators between stressful life events and the wish to die, taking into consideration the effect of gender as well.

## Methods

### Participants

In total, 1,020 doctors and resident doctors working in France took part in this study from March to September 2018. The sample includes participants from diverse medical specialties and practice modes, such as those involved in internships, freelancing, and working in both private and public hospitals. We did not apply any exclusion criteria. Several organizations, including resident associations in France, regional unions for health professionals, and regional councils of the college of physicians, played a role in facilitating communication with participants by sending them email invitations. While not all organizations agreed to send the invitation, those that did represent a collective total of more than 30,000 physicians in France. All the physicians who agreed to participate in the survey, received a link to a Google Form and answered a brief online questionnaire (see Appendix 1). Answers to the questionnaire were completely anonymous.

The physicians in our study were mainly women (*n* = 634, 62.2%) aged 38.0 years on average (±13.6; Table 1). Of these, 41.7% were residents and 58.3% were senior physicians. Over half of the sample comprised general practitioners (*n* = 581, 57%), while surgical specialists accounted for 6.8% (*n* = 69), and medical specialists constituted 36.3% (*n* = 370).

**Table 1:** Wish to die according to demographics, professional status and personality.

Categorical Covariates		Wish to die		χ^2^/F	*p*
		YES *N* = 162 (%) or Mean ± SD	NO N = 858 (%) or Mean ± SD		
Female gender		111 (68.5)	523 (61)	3.31	0.069
Age		37.86 ± 14.68	38.07 ± 13.33	0.032	0.857
Professional status	Resident	79 (48.8)	346 (40.3)	3.99	**0.046**
	Senior	83 (51.2)	512 (59.7)		
Residents	General medicine	31 (39.2)	120 (34.7)	1.25	0.536
	Surgical specialty	5 (6.3)	34 (9.8)		
	Medical specialty	43 (54.4)	192 (55.5)		
Seniors	General medicine	62 (74.7)	368 (71.9)	3.72	0.156
	Surgical specialty	7 (8.4)	23 (4.5)		
	Medical specialty	14 (16.9)	121 (23.6)		
Type of medical practice (seniors)	Private	68 (81.9)	447 (87.3)	1.78	0.183
	Public	15 (18.1)	65 (12.7)		
Personality traits	Emotional stability	3.75 ± 1.07	4.42 ± 1.19	44.52	**<0.001**
	Extraversion	4.48 ± 1.34	4.59 ± 1.17	1.21	0.271
	Openness	4.68 ± 1.20	4.53 ± 1.14	2.33	0.127
	Agreeableness	5.16 ± 0.92	5.28 ± 0.89	2.47	0.116
	Conscientiousness	5.15 ± 1.01	5.26 ± 0.88	2.43	0.119

SD, standard deviation; χ^2^, chi-square test; F, T-test statistic.Bold values which are significative.

The authors assert that all procedures contributing to this work comply with the ethical standards of the relevant national and institutional committees on human experimentation and with the Helsinki Declaration of 1975, as revised in 2008.

### Assessment

The questionnaire comprised several sections, namely: basic demographic data (sex, age, medical specialty and mode of medical practice), exposure to stressful events (personal and work-related), personality traits, a single-item assessment of burnout and a single question about the wish to die. Participants could answer “yes” or “no” to the following question: “in the last 12 months, have you ever wanted to be dead or wanted to sleep and never wake up?.” We aimed to refrain from using the term “suicide” and opted for an indirect approach to mitigate the emotional sensitivities associated with the term. To ensure the completeness of data, participants had to provide an answer for each question before moving on to the next one. Many validated scales of suicidal risk in the literature are relatively long to complete. In our study the questionnaire was deliberately built in a short form in order to reinforce participation in a population where significant time constraints are generally experienced (estimated completion time 2 to 5 min).

Exposure to stressful events over the past 12 months was evaluated via two closed-ended questions, one about work-related stressful events (“Have you experienced one (or more) stressful events related to the practice of medicine?”), and the other about stressful personal events (“Have you experienced one (or more) stressful events outside your medical practice?”). The term “stressful event” was defined in the questionnaire as “an event that has a sufficiently stressful impact to exceed a person’s capacity to adapt to the situation.” Whenever the answer was “Yes,” participants had to answer a multiple-choice question about the nature of that event. The list of stressful events was constructed based on the List of Threatening Experiences (47, 48) and the Social Readjustment Rating Scale (49), which includes a list of life events with a social impact that may cause stress. An additional “Miscellaneous” option opened a free text field to describe any events that did not fit in with the list. Participants could choose an unlimited number of events. Subsequently, they described the impact of the event (or events) on their life according to its degree on a Likert scale (none/mild, moderate or severe/catastrophic).

Burnout was screened using the Single Maslach Burnout Inventory—Emotional Exhaustion, based on the Maslach Burnout Inventory (50). The validity of this questionnaire has been demonstrated (51) and already used for the medical population (52). Participants had to choose one item from among 5 graded items to best describe their current work-related feelings. Passive SI was assessed by a single closed-ended question concerning the wish to die: “In the last 12 months, have you ever wanted to die or go to sleep and never wake up?.” By using this single question, we aimed to maximize the acceptability of our questionnaire. The assessment of death desire for detecting suicidal risk was studied in 2011 (53) and showed an efficiency equivalent to the questions used to screen for SI.

Personality was evaluated via the short version of the Big Five Inventory questionnaire (BFI-S), a short version of the Big-Five Personality questionnaire (54, 55). The BFI-S includes 15 items and participants must report their level of agreement with each one on a Likert scale from 1 to 7 (in which 1 = “strongly disagree” and 7 = “totally agree”). The BFI-S scores provide information regarding the intensity of five personality traits: emotional stability (neuroticism), extraversion, openness, agreeableness and conscientiousness. This questionnaire included the main personalities that have been considered to have an impact on physicians’ feeling of well-being at work and the risk of suicide.

### Statistical analysis

First, a descriptive analysis was performed to characterize our sample by means of univariate analyses. Chi-squared tests were employed for categorical variables and T-tests for continuous ones to compare the presence or absence of wish to die for each covariate.

Secondly, logistic regression analyses were conducted with all predictor variables, excluding the type of practice, to examine the influence of personality and gender on the relationship between stressful events and the desire to die. The type of practice variable (private/public) had no discernible effect on the wish to die. Since it could not be applied to residents, we deliberately chose to exclude it. Including this variable did not alter the results.

Thirdly, separate moderated moderation analyses were performed to examine the effect of each personality trait and gender on the relationship between stressful events and the wish to die. The analyses were conducted using the Hayes PROCESS macro with the Model 3 function and a bootstrapping approach comprising 5,000 bootstrap moderated regressions. “Impact of work-related stressful event(s)” or “impact of personal stressful event(s)” was used as a predictive variable, while “wish to die” served as the dependent variable. Personality trait scores were included as primary moderators (W), and gender (women/men) was considered a secondary moderator (Z). Professional status (resident/senior) and age were used as covariates, as they demonstrated significant associations with the wish to die in the logistic regression model. Burnout was not included due to its high collinearity with work-related stressful events (*p* < 0.001). Personality trait levels were categorized into three groups based on standard deviations: below the mean (−1 SD), at the mean, and above the mean (+1 SD). The Johnson-Neyman analysis was employed to identify the region of significance for the conditional effect of personality trait levels, contingent upon gender.

All statistical analyses were carried out using SPSS v.24 software. The significance threshold was set at *p* < 0.05.

## Results

A large majority of the study participants (n = 769, 75.4%) reported exposure to a work-related stressful event in the previous 12 months (Table 2). The most common work-related stressful events were: (1) exposure to “work overload” (*n* = 385, 51.3%), (2) conflicts or complaints from a patient and/or family (*n* = 286, 37.1%), and (3) verbal or physical aggression while at work (*n* = 251, 32.7%). Nearly half (*n* = 318, 41.4%) the participants who had been exposed to work-related stressful events considered their impact as severe/catastrophic. Burnout was screened as positive in 25.6% of participants (*n* = 261).

**Table 2:** Wish to die according to the exposure to stressful events.

Exposure to stressful events	Wish to die		χ^2^	*p*
	YES *N* = 162 (%)	NO *N* = 858 (%)		
Total number of events	3.12 ± 0.15	2.02 ± 0.06	55.280	**<0.001**
Work-related stressful events	146 (90.1)	623 (72.2)	22.527	**<0.001**
^†^Severe/catastrophic impact	74 (76.3)	348 (65.8)	9.219	**0.010**
Assaults/threats at workplace	49 (30.2)	202 (23.5)	0.070	0.792
Conflict/claim/complain from patient and/or family	59 (36.4)	227 (26.5)	0.800	0.371
Harassment	12 (7.4)	14 (1.6)	12.913	**<0.001**
Adverse event related to care	44 (27.2)	171 (19.9)	0.425	0.515
Conflict with a colleague/superior	57 (35.2)	144 (16.8)	15.542	**<0.001**
Conflict with the administrative hierarchy	33 (20.4)	71 (8.3)	12.701	**<0.001**
Change of position/increased responsibilities	29 (17.9)	108 (12.6)	0.516	0.473
Work overload	80 (49.4)	315 (36.7)	0.848	0.357
Number of events	2.24 ± 0.13	1.45 ± 0.05	42.74	**<0.001**
Personal stressful events	100 (61.7)	420 (49)	8.902	**0.003**
^†^Severe/catastrophic impact	43 (84.3)	243 (74.3)	4.392	0.111
Serious illness/injury/assault	16 (9.9)	52 (6.1)	0.931	0.335
Serious illness/injury/assault of a loved one	23 (14.4)	127 (14.8)	2.062	0.151
Death of a loved one	22 (13.6)	89 (10.4)	0.032	0.859
Separation of the couple	16 (9.9)	43 (5)	2.666	0.103
End of a lasting relationship	9 (5.6)	15 (1.7)	5.407	**0.020**
Serious problems with a loved one or a neighbor	15 (9.3)	40 (4.7)	2.561	0.110
Financial problems	24 (14.8)	41 (4.8)	14.970	**<0.001**
Loss/theft of valuables	4 (2.5)	12 (1.4)	0.354	0.552
Wedding	4 (2.5)	13 (1.5)	0.209	0.647
Arrival of a new child	7 (4.3)	46 (5.4)	1.378	0.240
Number of events	0.88 ± 0.08	0.56 ± 0.03	20.02	**<0.001**

^†^Severity categories: None/mild, Moderate, Severe/catastrophic. Data from continuous variables are mean and standard error of the mean.Bold values which are significative.

One in two participants (*n* = 520, 51%) had experienced recent personal stressful events unrelated to their work, and half of them reported that these events had a severe impact on their life (*n* = 280, 27.4%). One in six physicians had endorsed a wish to die in the previous 12 months (*n* = 162, 15.9%). In terms of personality, agreeableness (*n* = 433, 42.5%) and conscientiousness (*n* = 378, 37.1%) were the most common dominant traits among the participants (Table 1).

### Comparison by presence of death desire

In our study, we found no significant difference in the prevalence of wish to die according to gender, age, type of practice, or medical specialty (Table 1). Henceforth, we present only significant results unless otherwise specified. Residents were more likely to endorse a wish to die than seniors (*p* = 0.046). Regarding personality traits, the sole noteworthy distinction revolved around the trait of emotional stability, which exhibited lower levels among physicians endorsing a wish to die compared to the rest of the sample (3.75 ± 1.07 vs. 4.42 ± 1.19; *p* < 0.001).

Among participants reporting a wish to die, 90.1% had recently experienced at least one work-related stressful event and only 9.9% had not (*p* < 0.001). Specifically, a high percentage of participants (*n* = 466, 45.7%) who wished to die endorsed a severe to catastrophic stressful event on their life (*p* = 0.010). All types of work-related stressful events except two (“assaults/threats at the workplace” and “change of position/increased responsibilities”) were significantly associated with the wish to die (Table 2). Physicians in burnout were more likely to endorse a wish to die (*p* < 0.001; Table 3). Almost half of burnt-out physicians reported a wish to die (*n* = 80, 49.4%), compared to one in five for those who were not (*n* = 181; 21.1%).

**Table 3:** Wish to die according to answers to the Single Maslach Burnout Inventory—Emotional Exhaustion questionnaire (MBI-EE).

Burnout	Desire for death		χ^2^	df	*p*
	YES *N* = 162 (%)	NO *N* = 858 (%)			
Burnout (Yes)	80 (49.4)	181 (21.1)	57.27	1	**<0.001**
No burnout (Item 1) *N* = 254	10 (3.9)	244 (96.1)	80.05	4	**<0.001**
No burnout (Item2) *N* = 505	72 (14.3)	433 (85.7)			
Severity 1 (Item 3) *N* = 200	54 (27)	146 (73)			
Severity 2 (Item 4) *N* = 24	9 (37.5)	15 (62.5)			
Severity 3 (Item 5) *N* = 37	17 (45.9)	20 (54.1)			

Bold values which are significative.

Most participants who reported a wish to die (*n* = 629, 61.7%) had also experienced recent personal stressful events (*p* = 0.003). Among them, 26.5% estimated the impact on their life as severe to catastrophic. Certain types of events were significantly associated with the wish to die, namely: “end of a lasting relationship,” “financial problems,” “separation of the couple,” and “serious problems with a loved one or a neighbor” (Table 2).

### Logistic regression

The logistic regression model with all predictor variables (Table 4) reveals that advancing age (OR = 1.03; 95%CI = 1.01–1.05; *p* = 0.010), being in training as a resident rather than a senior physician (OR = 2.21; 95%CI = 1.24–3.95; *p* = 0.007), experiencing a work-related stressful event (OR = 2.18; 95%CI = 1.24–3.84; *p* = 0.007), and a positive burnout screen (OR = 2.66; 95%CI = 1.82–3.88; *p* < 0.001) are independently associated with a desire to die in the prior 12 months. Conversely, each incremental point in emotional stability predicts a reduced risk of desiring to die (OR = 0.69; 95%CI = 0.69–0.82; *p* < 0.001).

**Table 4:** Logistic regression model on wish to die according to clinical features, life events and personality traits.

Variables	B	*p*-value	OR	95% CI	
				Lower	Upper
Gender	0.168	0.427	1.183	0.781	1.794
Age	0.024	**0.022**	1.025	1.004	1.046
Professional Status (resident/senior)	0.793	**0.007**	2.210	1.237	3.947
**Specialty (reference: GP)**					
Surgical specialty	−0.081	0.825	0.922	0.450	1.889
Medical specialty	−0.258	0.224	0.772	0.509	1.172
Work-related stressful event (y/n)	0.782	**0.007**	2.185	1.240	3.849
Burnout (y/n)	0.977	**0.000**	2.657	1.821	3.877
Personal stressful event (y/n)	0.158	0.406	1.171	0.806	1.702
Emotional Stability	−0.365	**0.000**	0.694	0.586	0.822
Extraversion	−0.018	0.817	0.982	0.843	1.144
Openness	0.133	0.113	1.143	0.969	1.348
Agreeableness	−0.060	0.559	0.942	0.769	1.153
Conscientiousness	−0.170	0.103	0.844	0.688	1.035

CI, Confidence Interval; OR, Odds ratio; Degrees of freedom, 1. Specialty values: general medicine, surgical specialty, and medical specialty. Categorical values are shown in brackets.Bold values which are significative.

### Moderated regressions

**Figure 1:**
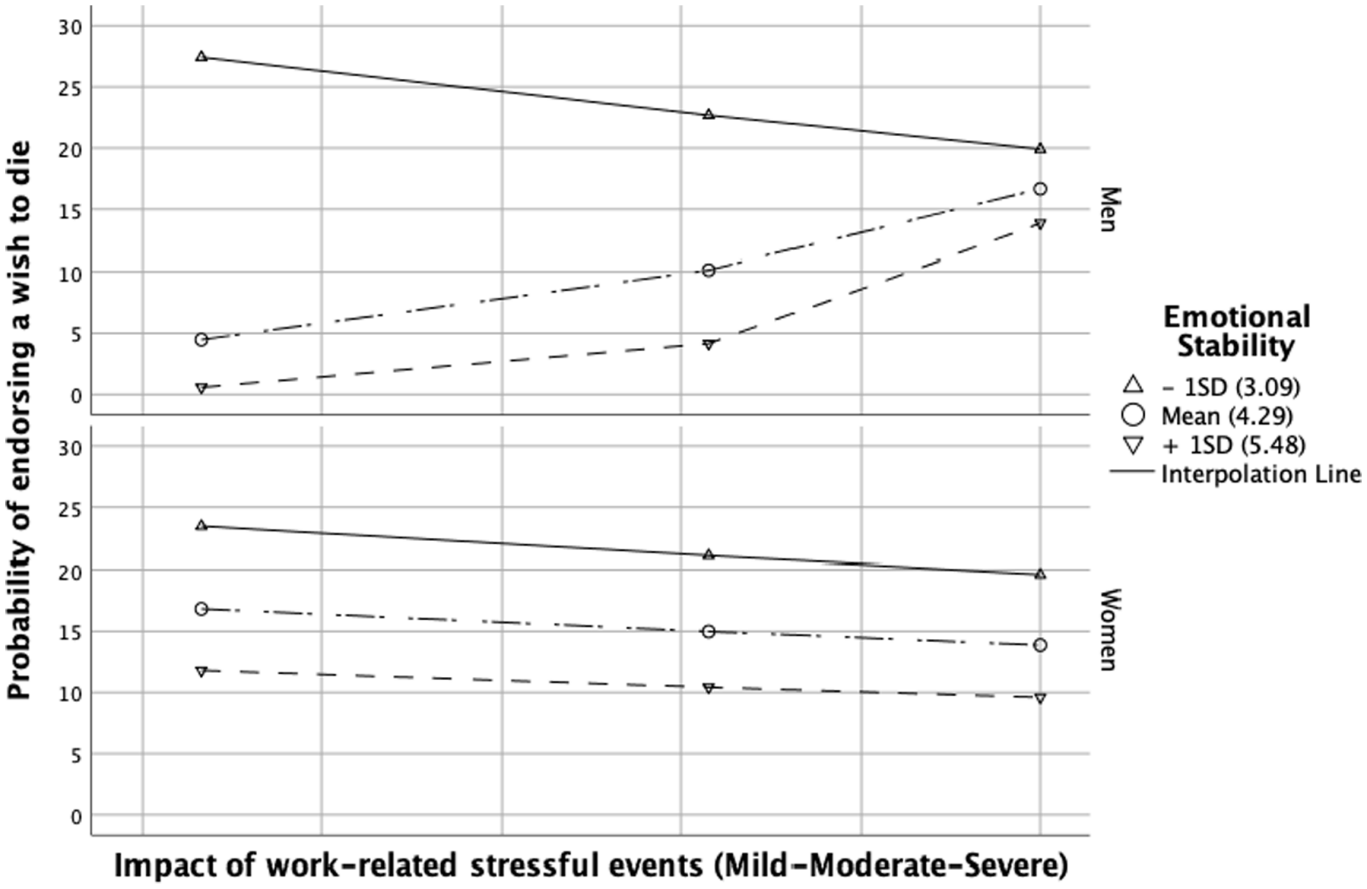
Conditional effect of the impact of work-related stressful event(s) on wish to die depending on the level of emotional stability. Conditioning values are at minus one standard deviation (SD), the mean, and plus one SD.

Separated moderation analyses on the impact of stressful events were carried out for each personality trait. Coefficients for significant results can be found in Supplementary Tables 1–3. Emotional stability and extraversion had a significant moderating effect on the relationship between the impact of work-related stressful events and the wish to die. The interaction term between work-related stressful events and emotional stability had a positive significant effect on wish to die (3.34, 95% CI = [0.62–3.94], *p* < 0.007). There was also a significant effect of the three-way interaction “impact of work-related stressful events” + “emotional stability” + “gender” (γ^2^ = 9.07, *p* = 0.003). Johnson-Neyman analysis revealed that, moderate (Effect = 1.55, *p* = 0.025) or high (Effect = 3.54, *p* = 0.008) emotional stability enhanced the impact of work-related stressful events on the likelihood of wishing to die in men, but not in women. Graphical probing was performed to understand the trend of the moderated moderation effect (see Figure 1).

**Figure 2:**
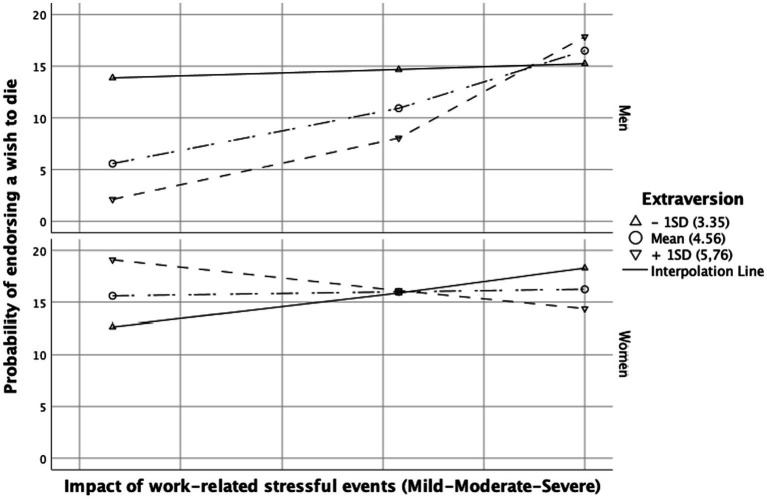
Conditional effect of the impact of work-related stressful event(s) on wish to die depending on the level of extraversion. Conditioning values are at minus one standard deviation (SD), the mean, and plus one SD.

The interaction term between work-related stressful events and extraversion had also a positive significant effect on wish to die (2.28, 95% CI = [0.91–5.77], *p* = 0.007). There was a significant effect of the three-way interaction “impact of work-related stressful events” + “extraversion” + “gender” (γ^2^ = 9.74, *p* = 0.002). Johnson-Neyman analysis revealed that, moderate (Effect = 1.29, *p* = 0.020) or high (Effect = 2.47, *p* = 0.011) extraversion increased the impact of work-related stressful events on the likelihood of wishing to die in men, but not in women. This effect can be observed in Figure 2.

**Figure 3:**
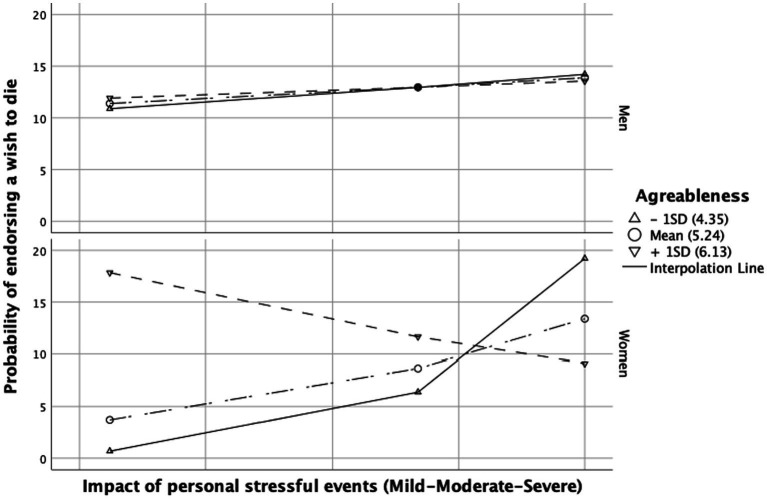
Conditional effect of the impact of personal stressful event(s) on wish to die depending on the level of agreeableness. Conditioning values are at minus one standard deviation (SD), the mean, and plus one SD.

The effect of personal stressful events on the probability of endorsing a wish to die was found to be only partially moderated by agreeableness. Although with a non-significant trend, the interaction term between personal stressful events and agreeableness had a positive effect on wish to die (2.99, 95% CI = [−0.41–6.39], *p* = 0.085). There was a significant effect of the three-way interaction “impact of personal stressful events” + “agreeableness” + “gender” (γ^2^ = 7.22, *p* = 0.007). Johnson-Neyman analysis revealed that low agreeableness enhanced the impact of personal stressful events on the likelihood of wishing to die in women only (Effect = 4.72, *p* = 0.011), not in men. This effect can be observed in Figure 3.

## Discussion

In our study, one in six physicians (15.9%) had endorsed a wish to die in the 12 months preceding the survey. A quite similar prevalence of SI (13%) had been found in a study among French general practitioners (56). This lends support to the viability of utilizing a single question on the wish to die as a screening instrument for SI among physicians, as found in the literature (53). Nevertheless, other studies have found a prevalence of SI varying from 11% to 50% (6, 8, 12–15). Indeed, some studies have found a higher prevalence of SI than in our study but these consider lifetime prevalence rather than prevalence over the previous year. The variability in prevalence and the comparatively lower range observed in our study may be explained by the majority of respondents being senior physicians (58.3%), with the wish to die statistically more associated with residents than seniors. Prior studies have already reported a higher suicide risk during residency which declined with the physicians’ experience and changing lifestyles (2, 43). Finally, with regard to the gender distribution in our sample, it closely mirrors that of the broader physician population, encompassing approximately 60% women. Additionally, our sample exhibits a slightly younger age profile, averaging around 40 years, compared to the reference population’s average age of 50 years (data sourced from the French Medical Association). These demographic variations do not account for the observed low prevalence of SI in our sample.

A notable 25.6% of participants in our sample reported experiencing burnout within the past 12 months but the prevalence of burnout in other studies is even higher (7, 23, 25, 57, 58). For instance, a systematic review and meta-analysis aggregated 26 studies including 4,664 residents of several disciplines and found a prevalence of burnout of 35.7% (57). Once more, most of these studies have focused only on medical students or have heterogeneous samples with medical students. Furthermore, certain medical specialties at a particular risk of burnout, like surgery (5) were under-represented in our sample. Last but not least, in our study, burnout was associated with wish to die and the impact seems to be severity-dependent. Today the subject of the complexity between burnout and depression is still under debate (2, 59, 60). The association between burnout and SI may be explained by the overlap of these concepts. Therefore, no adjustment was made for depression in considering the association between SI and burnout.

In our study, wish to die is particularly associated to stressful events during medical practice, often described as having a severe or catastrophic impact. In line with earlier studies, our findings show an association between work-related stressful events such as work overload, conflicts with colleagues and superiors (22) or administrative hierarchy and harassment (27) and the risk of experiencing a wish to die. Indeed, little participation in decisions about working life (little control over work-related stress factors) has already been identified as a powerful stress factor among physicians (61) and is associated with the risk of suicide (26). Moreover, we also found that the usual suicide risk factors like relational (62) and financial issues (63) were associated with the wish to die. These results argue for the external validity of our study and good representativity of our sample.

According to our results, the effect of stressors on wish to die was moderated both by personality traits and gender. Emotional stability, usually referred to as its counterpart neuroticism, was associated with wish to die specifically among physicians (43) and in the general population (34–37, 64–69). It constituted the sole personality trait linked to the presence of a wish to die in our sample, and this association was robust and independent of potential confounding factors. Overall, the higher the emotional stability, the lower the risk of endorsing a wish to die. Nevertheless, a closer examination through moderation analyses indicates that men with intermediate or high levels of emotional stability might exhibit a higher likelihood of endorsing a wish to die when exposed to moderate, and particularly severe, work-related stressful events. This finding is unexpected, as high levels of emotional stability are generally seen as a buffering factor against stressors in the workplace while low emotional stability has been linked to the perception of elevated job stress in young physicians (44). One interpretation could stem from the perceived greater significance of work status for men compared to women (70). Adverse life events in the workplace, coupled with the loss of work status, may have a more profound impact on men with high emotional stability who may not have anticipated such possibilities, intensifying the shock of the experience for them. However, for both genders, lower levels of emotional stability show higher probabilities of endorsing a wish to die when confronted with work-related stressors of comparable severity (Figure 1). There is evidence supporting the influence of low emotional stability on an increased risk of suicide when individuals are exposed to stress factors (71), but also conflicting perspectives. Notably, one study identified low emotional stability as a potential protective factor even after adjusting for depression (37). Psychiatric comorbidities could also introduce bias to the data concerning this trait (72).

In our findings, men with high and intermediate levels of extraversion, when exposed to work-related stressful events, were more prone to expressing a wish to die. The results concerning women were not significant. This observation converges with the conclusions of one study in favor of an association between extraversion and SI (36, 37), but to date, to our knowledge, no study has examined this variable as a moderator of the impact of work stressors on SI. More broadly, one study was able to hypothesize that the personality trait of extraversion may intensify the impact of low social support on suicide risk (73). The relationship between extraversion and suicide risk remains a subject of debate, with conflicting or non-significant data in the existing literature (66–69, 74–77). More research is needed on this association.

Finally, female physicians with low levels of agreeableness were more likely to endorse a wish to die when exposed to adverse personal events. These results confirm previous data about the protective role of agreeableness against SI or the risk of suicide (34–36, 66). Low levels of agreeableness in interpersonal relationships might be associated with poor social outcomes due to inadequate social support (78). In turn, low social support has been associated with well-being (45) and suicide risk, both in general and in the workplace (22). For men, the lack of significant findings might be related to the smaller sample size, as well as a differential impact of the trait agreeableness on both social and professional outcomes compared to women. It has been shown that men with high agreeableness tend to achieve less favorable outcomes professionally (79). It should be also noted that female physicians are more often associated with the risk of suicide than men (4, 10). Notably, 21.2% of women in our sample expressed a wish to die, in contrast to 15.2% of men.

## Limitation

Although the assessment of wish to die has been shown to be an effective means of assessing SI, it remains an indirect proxy for suicidal risk. Moreover, because the questionnaire only included a single question on the presence or absence of SI, we lack information on the frequency and intensity of these ideas. Future studies could assess SI more closely and may adjust their results on these variables. Thirdly, our study is also limited by the impossibility to control for risk factors associated with suicide like depressive symptoms, addictive behaviors or other psychiatric comorbidities. Finally, only physicians from the French population were studied, limiting the generalization of results to other countries. Recent studies have shown the possibility of extracting large population data on SI from social networks and analyze it using artificial intelligence for similar research objectives (80, 81).

However, certain strengths should also be mentioned. First, only a few studies have investigated the direct association between suicide risk and job stress (8, 43, 82, 83) or more precise work-related factors (22, 27, 84, 85) among physicians. Furthermore, there’s a scarcity of studies examining work-related factors in conjunction with personality traits (43), and to the best of our knowledge, few studies have investigated both, along with their interaction (71). Secondly, a substantial number of physicians participated in the survey and senior professionals were well-represented. This sample is reasonably reflective of the physician population in terms of gender and age. Thirdly, as our aim was to optimize the acceptability of the questionnaire, we were very careful not to mention the term “suicide” anywhere on it and suicidal risk was indirectly assessed by one single question about wish to die.

## Conclusion

In conclusion, our study revealed that approximately one in six physicians (equivalent to about one in five residents) reported a wish to die in the year preceding our investigations, and a quarter of them cited experiencing burnout. The desire to die among physicians is influenced by various factors, including personal stressful events (such as affective and financial issues) and specific work-related stressors like work overload, harassment, and interpersonal conflicts. Notably, certain personality traits—namely, emotional stability, extraversion, and agreeableness—appear to modify the impact of stressful events on the wish to die in certain scenarios. Emotional stability and extraversion levels moderate the influence of workplace stressors, while agreeableness moderates the effect of personal events. Consistent with existing literature, these findings underscore the significant concern about suicide risk among physicians and the noteworthy impact of work-related stressors. Nevertheless, further research is essential before targeted preventive strategies for the medical profession can be implemented.

## Data availability statement

The raw data supporting the conclusions of this article will be made available by the authors, without undue reservation.

## Ethics statement

The studies involving humans were approved by Institutional Review Board, Nimes University Hospital. The studies were conducted in accordance with the local legislation and institutional requirements. The participants provided their written informed consent to participate in this study.

## Author contributions

All authors listed have made a substantial, direct, and intellectual contribution to the work and approved it for publication.
