# Supplementary material for: Role of stressful life events and personality traits on the prevalence of wish to die among French physicians
Source: Front Public Health. 2024 Jan 23;12:1244605. doi: 10.3389/fpubh.2024.1244605 (PMC10844508; doi:10.3389/fpubh.2024.1244605)

Appendix 1: Online questionnaire

Age :

Sex : M/F

Profession (several possible answers) :

- Intern
- Freelance physician
- Hospital physician in the public sector
- Hospital physician in the private sector

Specialty : [Drop-down list of specialties]

*** Over the last 12 months have you experienced one (or several) stressful events* related to your job as a doctor ?

- Yes
- No

  *"Stressful event" refers to any event that has a sufficiently stressful impact to exceed a person's capacity to adapt to the situation. Please note that stress may be the consequence of negative events as well as positive ones.

If so, what type of event/events (several possible answers) ? :

- Assault (physical and/or verbal) in the context of work
- Conflicts /Claims / Complaints or threats to complain from patients and/or the patient's family
- Harassment at work
- Adverse events related to care (medical error) whatever the consequences
- Conflict with a colleague/or superior at work
- Conflict with the administrative hierarchy

- Change of position and/or new responsibilities
- Work overload
- Miscellaneous: [free comments section]

What effect did this event/these events have on you ?

- None
- Slight
- Moderate
- Serious
- Catastrophic
- Don't know / No comment

*** Over the last 12 months have you experienced one (or several) stressful events* unrelated to your job as a doctor ?

- Yes
- No

*"Stressful event" refers to any event that has a sufficiently stressful impact to exceed a person's capacity to adapt to the situation. Please note that stress may be the consequence of negative events as well as positive ones.

If so, what type of events (several possible answers)? :

- Serious illness/ injury / assault
- Serious illness / injury / assault of a loved-one
- Death of a loved one
- Divorce/separation
- Unemployment
- End of a stable relationship

- Serious problem with a relative or a neighbor
- Financial difficulties
- Problems with the law
- Loss / Theft of a valuable object
- Wedding
- Arrival of a new baby
- Miscellaneous : [free comments section]

What effect did this event/these events have on you ?

- None
- Slight
- Moderate
- Serious
- Catastrophic
- Don’t know / No comment

*** Over the last 12 months have you ever wanted to die or go to sleep and never wake up again ?

- Yes
- No

Appendix 2: Number of adherents for each organization contacted

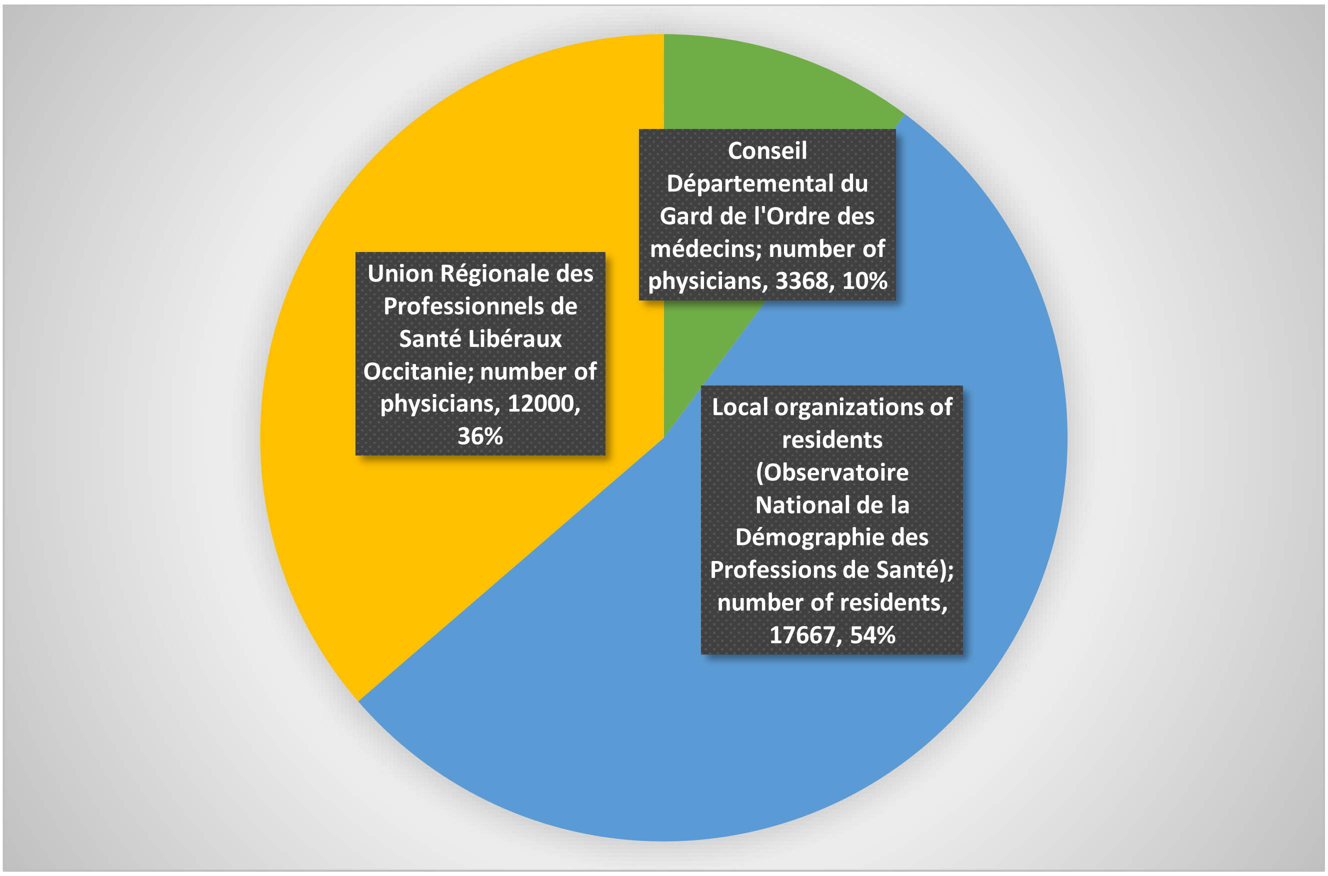

Supplement: Supplementary file 1 [file Data_Sheet_1.pdf]
