# Supplementary material for: Role of stressful life events and personality traits on the prevalence of wish to die among French physicians
Source: Front Public Health. 2024 Jan 23;12:1244605. doi: 10.3389/fpubh.2024.1244605 (PMC10844508; doi:10.3389/fpubh.2024.1244605)
Supplement: Supplementary file 2 [file Table_1.docx]

**Supplementary Table 1:** Moderation model for presence of wish to die with the impact of work-related stressful events as predictor, and emotional stability and gender as moderators.

|  | **Coefficient (95 % CI)** | **p-value** |
| --- | --- | --- |
| Impact of work-related stressful events | -10.99 (-19.53–(-2.45)) | **.012** |
| Emotional stability | -10.05 (-17.11–(-2.99)) | **.005** |
| Interaction 1 | 3.34 (.91–5.77) | **.007** |
| Gender | -15.64 (-28.72–(-2.56)) | **.019** |
| Interaction 2 | 5.38 (.77–9.98) | **.022** |
| Interaction 3 | 4.85 (1.14–8.56) | **.010** |
| Interaction 4 | -1.67 (-2.95–(-.39)) | **.011** |
| Age† | .034 (.00–.06) | **.017** |
| Professional Status† | -1.06 (-1.78–(-.34)) | **.004** |

^†^ Covariates

Results are expressed in a log-odds metric.

**Product terms key:** Interaction 1: Impact of work-related stress X Emotional Stability; Interaction 2: Impact of work-related stress X Gender; Interaction 3: Emotional Stability X Gender; Interaction 4: Impact of work-related stress X Emotional Stability X Gender.

**Supplementary Table 2:** Moderation model for presence of wish to die with the impact of work-related stressful events as predictor, and extraversion and gender as moderators.

|  | **Coefficient (95 % CI)** | **p-value** |
| --- | --- | --- |
| Impact of work-related stressful events | -7.88 (-14.23–(-1.53)) | **.015** |
| Extraversion | -6.58 (-11.29–(-1.87)) | **.006** |
| Interaction 1 | 2.28 (.62–3.94) | **.007** |
| Gender | -13.36 (-23.52–(-3.20)) | **.010** |
| Interaction 2 | 4.75 (1.09–8.41) | **.011** |
| Interaction 3 | 3.75 (1.21–6.28) | **.004** |
| Interaction 4 | -1.31 (-2.21–(-.41)) | **.004** |
| Age† | .030 (.00–.06) | **.035** |
| Professional Status† | -1.04 (-1.76–(-.32)) | **.005** |

^†^ Covariates

Results are expressed in a log-odds metric.

**Product terms key:** Interaction 1: Impact of work-related stress X Extraversion; Interaction 2: Impact of work-related stress X Gender; Interaction 3: Extraversion X Gender; Interaction 4: Impact of work-related stress X Extraversion X Gender.

**Supplementary Table 3:** Moderation model for presence of wish to die with the impact of personal stressful events as predictor, and agreeableness and gender as moderators.

|  | **Coefficient (95 % CI)** | **p-value** |
| --- | --- | --- |
| Impact of personal stressful events | -16.92 (-36.01–2.17) | .082 |
| Agreeableness | -8.54 (-18.35–1.25) | .087 |
| Interaction 1 | 2.99 (-.41–6.39) | .085 |
| Gender | -51.15 (-93.44–(-8.86)) | **.018** |
| Interaction 2 | 17.83 (3.44–32.21) | **.015** |
| Interaction 3 | 8.86 (1.68–16.05) | **.015** |
| Interaction 4 | -3.11 (-5.56–(-.65)) | **.013** |
| Age† | .031 (-.00–.06) | .082 |
| Professional Status† | -.65 (-1.61–.31) | .184 |

^†^ Covariates

Results are expressed in a log-odds metric.

**Product terms key:** Interaction 1: Impact of personal stress X Agreeableness; Interaction 2: Impact of personal stress X Gender; Interaction 3: Agreeableness X Gender; Interaction 4: Impact of personal stress X Agreeableness X Gender.
